# Autonomic cardiovascular alterations as therapeutic targets in chronic kidney disease

**DOI:** 10.1007/s10286-021-00786-6

**Published:** 2021-02-19

**Authors:** Gino Seravalle, Fosca Quarti-Trevano, Jennifer Vanoli, Chiara Lovati, Guido Grassi

**Affiliations:** 1 Clinica Medica, Department of Medicine and Surgery, University Milano-Bicocca, Milan, Italy; 2 Clinica Medica, University Milano-Bicocca, Via Pergolesi 33, 20052 Monza, Italy

**Keywords:** Autonomic nervous system, Sympathetic activity, Parasympathetic activity, Microneurography, Chronic renal failure, Dialysis, Kidney transplantation, Renal denervation, Carotid baroreceptor stimulation

## Abstract

**Purpose:**

The present paper will review the impact of different therapeutic interventions on the autonomic dysfunction characterizing chronic renal failure.

**Methods:**

We reviewed the results of the studies carried out in the last few years examining the effects of standard pharmacologic treatment, hemodialysis, kidney transplantation, renal nerve ablation and carotid baroreceptor stimulation on parasympathetic and sympathetic control of the cardiovascular system in patients with renal failure.

**Results:**

Drugs acting on the renin–angiotensin system as well as central sympatholytic agents have been documented to improve autonomic cardiovascular control. This has also been shown for hemodialysis, although with more heterogeneous results related to the type of dialytic procedure adopted. Kidney transplantation, in contrast, particularly when performed together with the surgical removal of the native diseased kidneys, has been shown to cause profound sympathoinhibitory effects. Finally, a small amount of promising data are available on the potential favorable autonomic effects (particularly the sympathetic ones) of renal nerve ablation and carotid baroreceptor stimulation in chronic kidney disease.

**Conclusions:**

Further studies are needed to clarify several aspects of the autonomic responses to therapeutic interventions in chronic renal disease. These include (1) the potential to normalize sympathetic activity in uremic patients by the various therapeutic approaches and (2) the definition of the degree of sympathetic deactivation to be achieved during treatment.

## Introduction

Chronic kidney disease is characterized by profound alterations in the autonomic control of the cardiovascular system. These include (1) pronounced activation of sympathetic cardiovascular effects, with evidence of important regional differentiation, particularly at the level of the kidneys [1, 2], (2) the early occurrence of adrenergic abnormalities in the clinical course of the disease, with direct proportionality to the severity of the renal dysfunction [3–5], (3) a reduction in the vagal inhibitory influence on sinus node, resulting in an increase in resting heart rate values [6], (4) impaired modulation of both vagal and sympathetic cardiovascular effects exerted by the arterial baroreceptors [3–6], (5) impaired cardiopulmonary receptor control of sympathetic vasoconstrictor tone and renin release from the juxtaglomerular cells [3–6], (6) chemoreflex activation [6] and (7) reduced sensitivity of the alpha adrenergic vascular receptors [6]. It has also been suggested that, similarly to what happens in congestive heart failure, in the initial phases of kidney disease, the autonomic changes (particularly the sympathetic ones) may have a compensatory function, guaranteeing renal perfusion and thus a normal or pseudo-normal glomerular filtration rate [7]. However, the autonomic alterations described in renal failure and aggravated by the presence of diabetes and obesity, which represent major contributors to the occurrence of renal disease [8], may over time exert an adverse clinical impact favoring the development and progression of cardiovascular complications, end-organ damage and life-threatening cardiac arrhythmias [3, 7–11]. This may represent the pathophysiological background for the finding that both parasympathetic and sympathetic alterations bear a specific clinical relevance for determining patients’ prognosis, even when analyzed data are adjusted for confounders [10, 12–14].

The present paper will review the impact of the therapeutic approaches employed in the management of renal failure on the autonomic dysfunction characterizing the disease. This will be done first by discussing the autonomic effects of cardiovascular drugs in patients with renal failure. We will then examine the impact of different types of dialytic procedures as well as renal transplantation on autonomic cardiovascular control. Emphasis will be given to the autonomic effects of procedural interventions such as carotid baroreceptor stimulation and renal nerve ablation in chronic renal failure. The paper will then discuss three final issues: first, the relevance of the heart-kidney crosstalk as therapeutic targets in kidney disease; second, whether and to what extent the therapeutic interventions mentioned above may be capable of restoring the autonomic function in chronic kidney disease to physiological levels; and finally, the optimal level of sympathetic drive to be achieved during the therapeutic intervention (drugs, hemodialysis, kidney transplantation, renal denervation and perhaps baroreflex activation therapy). These questions may have important clinical implications, given the already mentioned unfavorable impact of autonomic dysfunction on patient prognosis.

### Autonomic effects of cardiovascular drugs in chronic kidney disease

Drugs currently used in the treatment of patients with chronic kidney disease are aimed at exerting direct and indirect (i.e. blood pressure reduction-dependent) nephroprotective effects to limit the progression of the kidney dysfunction and control the elevated blood pressure values almost invariably accompanying advanced renal failure [15]. They are also aimed, however, at exerting favorable effects on autonomic function [3, 6, 7]. As far as parasympathetic alterations are concerned, evidence has been provided that some drugs may improve vagal control of the heart rate, as assessed via power spectral analysis of the heart rate signal. This is the case for beta-blocking agents, for angiotensin II receptor antagonists and, although not always homogeneously, for angiotensin-converting enzyme (ACE) inhibitors [10, 16–18]. At variance from the effects on sympathetic cardiovascular drive (see below), statins failed to show any potentiating effect on vagal control of the heart, as assessed by heart rate variability, in patients with chronic renal failure [19].

The sympathetic overactivity typically displayed by patients with chronic kidney disease is favorably affected by drugs acting on the renin–angiotensin system. This is the case in particular for ACE inhibitors and angiotensin II receptor blockers, with acute intravenous administration and long-term oral administration having been shown to reduce the elevated values of efferent postganglionic muscle sympathetic nerve traffic directly recorded via the microneurographic technique [20, 21]. The effect was similar to that reported with the same drug classes in another clinical condition characterized by marked sympathetic overactivity, i.e. congestive heart failure [22]. Sympathoinhibitory effects have also been documented recently for renin inhibitors such as aliskiren, particularly when these drugs are administered in a therapeutic regimen which includes atorvastatin [23]. It should be emphasized that statins may play an important role in determining this effect, because a reduction in the elevated sympathetic nerve traffic values has been reported with these drugs even when given to patients without the concomitant administration of any other sympathomodulatory drug [24, 25]. Significant reductions in sympathetic cardiovascular drive, as assessed by the assay of venous plasma norepinephrine or more directly via the microneurographic technique, have been reported in chronic renal failure patients when treated with central sympatholytic agents such as clonidine and moxonidine, the latter drug being evaluated when administered on top of conventional treatment with pharmacologic compounds acting on the renin–angiotensin system [26–28]. It should be emphasized that the mechanisms responsible for the sympathomodulatory properties of the abovementioned drug classes appear to be multiple and heterogeneous, including (1) a reduction in the excitatory effects of angiotensin II on peripheral and central adrenergic neural drive, (2) a partial or full restoration of the sympathoinhibitory properties exerted by the arterial baroreflex and (3) a direct effect of the drugs (particularly but not exclusively central sympathoinhibitory drugs) on the central nervous system [7].

### Autonomic effects of the hemodialysis procedures

**Fig. 1:**
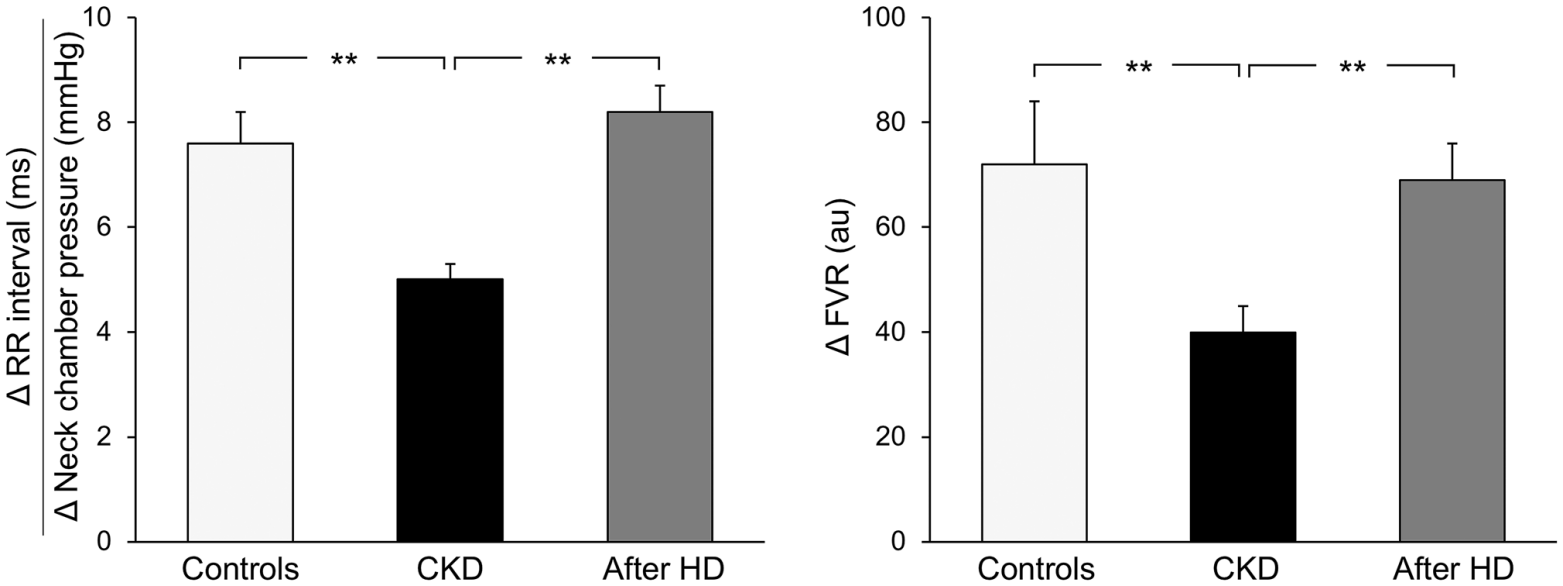
**Left panel** Baroreflex sensitivity values, derived from the ratio between R-R interval and neck chamber pressure changes, in healthy control subjects (controls, *n* = 10), in 25 patients with chronic kidney disease before (CKD) and after hemodialysis (after HD). **Right panel:** Reflex changes in forearm vascular resistance (FVR) in response to cardiopulmonary receptor deactivation in the same groups of subjects represented in the left panel. Data are shown as means ± SEM. Asterisks refer to the statistical significance between groups (**P < 0.01). Figure adapted from data in Ref. [35]

Whether and to what extent hemodialysis may allow the reversal and possible normalization of the altered autonomic profile of uremic patients remains a controversial issue. Indeed, some studies have documented an improvement in the parasympathetic control of the heart rate following acute hemodialysis, whereas others found no significant change [6, 29–33]. Our group investigated the vagal control of the heart rate exerted by carotid baroreceptors via the neck chamber technique in 25 young patients with chronic renal failure maintained on hemodialysis, before and after a single hemodialysis session, and in a group of age-matched healthy controls. In healthy subjects, graded carotid baroreceptor stimulation triggered a bradycardic response (assessed by electrocardiographic measurement tracing the lengthening of the R-R interval) that increased in magnitude as greater baroreflex stimulation was obtained [34]. This allowed us to assess the sensitivity of the baroreflex, which was, as expected, markedly reduced in the uremic patients as compared to age-matched healthy controls (Fig. 1, left panel). However, when assessed 2 h after the hemodialysis session, carotid baroreceptor function showed a significant improvement in uremic patients, with the average baroreflex sensitivity value becoming superposable to the one detected in healthy controls (Fig. 1, left panel). Additionally, in the same group of uremic patients, the acute hemodialysis procedure significantly improved the sensitivity of another reflexogenic area, i.e. the so-called cardiopulmonary volume-sensitive receptors, which physiologically regulate sympathetic vasoconstrictor tone in the forearm circulation. Cardiopulmonary volume-sensitive receptors were selectively deactivated by reducing venous return to the heart via a mild degree of lower body negative pressure. This allowed healthy controls to obtain a marked reflex increase in forearm vascular resistance, whose magnitude was significantly reduced in patients with chronic renal failure (Fig. 1, right panel). When the maneuver was repeated a few hours after hemodialysis, we noticed a significant improvement in the reflex forearm vascular responses, which became superposable in magnitude to those found in healthy controls (Fig. 1 right panels).

As noted above, some discrepancy exists between the results of the various published studies aimed at investigating the effects of the hemodialysis procedures on autonomic control of the cardiovascular system. The temporal duration of the uremic state, consistently different between the different published studies, may be one of the factors responsible, given the evidence that long-lasting uremia is associated with a neuropathy that is usually irreversible and unresponsive to hemodialysis [6]. Different degrees of volume overload between patients before the dialytic procedure may be another factor responsible for the different results reported in the various studies, knowing the relevance of central blood volume for determining reflex responses [35]. It should be emphasized, however, that the discrepancy in the findings between studies may also depend on the different types of dialytic procedure adopted in the various investigations. For example, it has been shown that peritoneal dialysis, at variance from hemofiltration and ultrafiltration, does not exert any major effect on autonomic alterations [6, 32, 36]. On the other hand, nocturnal hemodialysis and short-term hemodialysis sessions (maintaining the overall dialytic time duration unmodified during the week) may trigger favorable autonomic effects, improving arterial baroreflex sensitivity and exerting documented sympathoinhibitory effects [36–39].

### Autonomic function and kidney transplantation

**Fig. 2:**
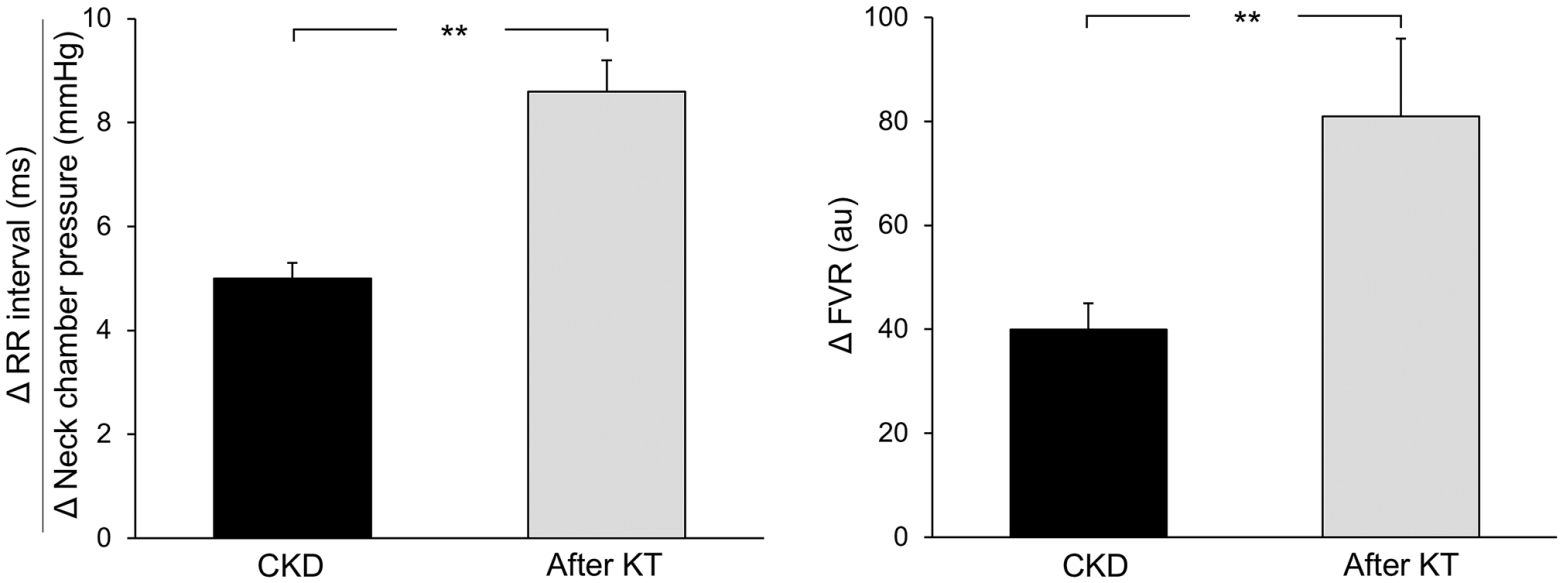
**Left panel** Baroreflex sensitivity values, derived from the ratio between R-R interval and neck chamber pressure changes, in nine patients with chronic kidney disease before (CKD) and after (after KT) kidney transplantation. **Right panel** Reflex changes in forearm vascular resistance (FVR) in response to cardiopulmonary receptor deactivation in the same groups of patients represented in the left panel. Data are shown as means ± SEM. Asterisks refer to the statistical significance between groups (**P < 0.01). Figure adapted from data in Ref. [35]

More homogeneous than those reported for hemodialysis appear to be the autonomic effects of kidney transplantation. This is the case for parasympathetic modulation of the heart, as assessed via the Valsalva maneuver, the expiration/inspiration ratio and more recently through assessment of heart rate variability via power spectral analysis of the heart rate signal [29, 37, 40]. Our group investigated the autonomic reflex effects of renal transplantation in nine uremic patients belonging to the original group of the 25 reported above [34]. The examination was carried out 3 months after kidney transplantation, and all transplanted patients were under immunosuppressive drug treatment. Patients underwent evaluation of heart rate-baroreflex sensitivity via the already mentioned neck chamber technique, and results obtained after kidney transplantation were compared to those obtained before the surgical procedure. As illustrated in Fig. 2, left panel, the sensitivity of the baroreflex, and thus the bradycardic response to baroreceptor stimulation, was significantly improved 3–6 months after renal transplantation, becoming almost superposable to that detected in healthy controls (see Fig. 1, left panel). Other investigators have similarly reported a potentiation of the baroreflex-heart rate reflex when assessed via vasoactive drug infusion in renal transplanted patients [4]. Taken together, these findings provide conclusive evidence of the occurrence of effective restoration of a major reflex mechanism involved in neurocirculatory regulation after renal transplantation.

Along with the baroreflex improvement, kidney transplantation additionally triggers a functional amelioration of the other major reflexogenic area involved in cardiovascular homeostatic control, i.e. the cardiopulmonary volume receptors. This was found in the nine transplanted patients we evaluated by examining the reflex forearm vascular responses to cardiopulmonary receptor deactivation via a mild degree of lower body negative pressure [34]. Results, illustrated in the right panel of Fig. 2, clearly show a significant potentiation of the forearm vasoconstrictor responses to the maneuver, making the values obtained almost superposable to those seen in age-matched healthy controls (Fig. 1, right panel).

In evaluating the autonomic effects of kidney transplantation, a crucial issue involves the analysis of the effects of the procedure on sympathetic neural cardiovascular function. This has been performed through the years by employing different methodologic approaches to assess noradrenergic function. By measuring venous plasma norepinephrine, our group and others found a significant reduction in sympathetic drive following renal transplantation [4, 6, 37]. This was also the case when ^123^metaiodobenzylguanidine imaging was employed to assess cardiac adrenergic innervation [41]. A further approach for assessing the noradrenergic effects of kidney transplantation is the microneurographic recording of efferent postganglionic muscle sympathetic nerve traffic. Quite surprisingly, the technique, which in several studies has enabled the detection of even small modifications in sympathetic drive, failed to show any significant reduction in adrenergic cardiovascular drive in renal transplanted patients [42]. This result is likely related to the finding that kidney transplant patients were under immunosuppressive drug treatment, which per se exerts marked sympathoexcitatory effects [43]. However, a further cause of sympathoexcitation was represented by the fact that initial evaluation of the muscle sympathetic nerve traffic was carried out in transplanted patients retaining the diseased native kidney(s) [42]. Indeed, surgical removal of the native kidneys combined with renal transplantation was shown in the same study to elicit an almost complete normalization of the sympathetic cardiovascular drive [38]. It is thus likely that signals arising from the diseased native kidney(s) and exerting effects on the central nervous system adrenergic activation may mask the true sympathoinhibitory effects of kidney transplantation in uremic patients [44].

### Autonomic effects of renal nerve ablation in chronic kidney disease

Although renal dysfunction represented a major exclusion criteria for patient recruitment in early clinical trials testing the efficacy and tolerability of percutaneous ablation of renal nerves in drug-resistant hypertension, more recent investigations have specifically evaluated the effects of the procedure in stage 3 and 4 chronic kidney disease. In general, the results of all the studies performed have provided clear-cut evidence that the procedure does not cause any detrimental effect on renal function [45]. Additionally, in studies with long-term follow-up, it was reported that the intervention slowed the progression of renal disease, with improvement, in some instances, of albumin and protein urinary excretion [45–48].

Whether and to what extent the outcome of renal denervation in chronic kidney disease depends on the direct (reduction in sympathetic drive) or indirect (blood pressure decrease) effects of the procedure remain undefined [49], also because the data on the sympathetic effects of renal denervation in chronic renal failure are scant. Indeed, only one study, carried out in nine patients with end-stage renal disease maintained on hemodialysis, has specifically investigated the muscle sympathetic nerve traffic responses to endovascular renal denervation [50]. Results of this proof-of-concept investigation documented the sympathoinhibitory effects of the procedure, with a significant reduction in sympathetic nerve traffic amounting on average to 20% during the 12-month follow-up.

**Fig. 3:**
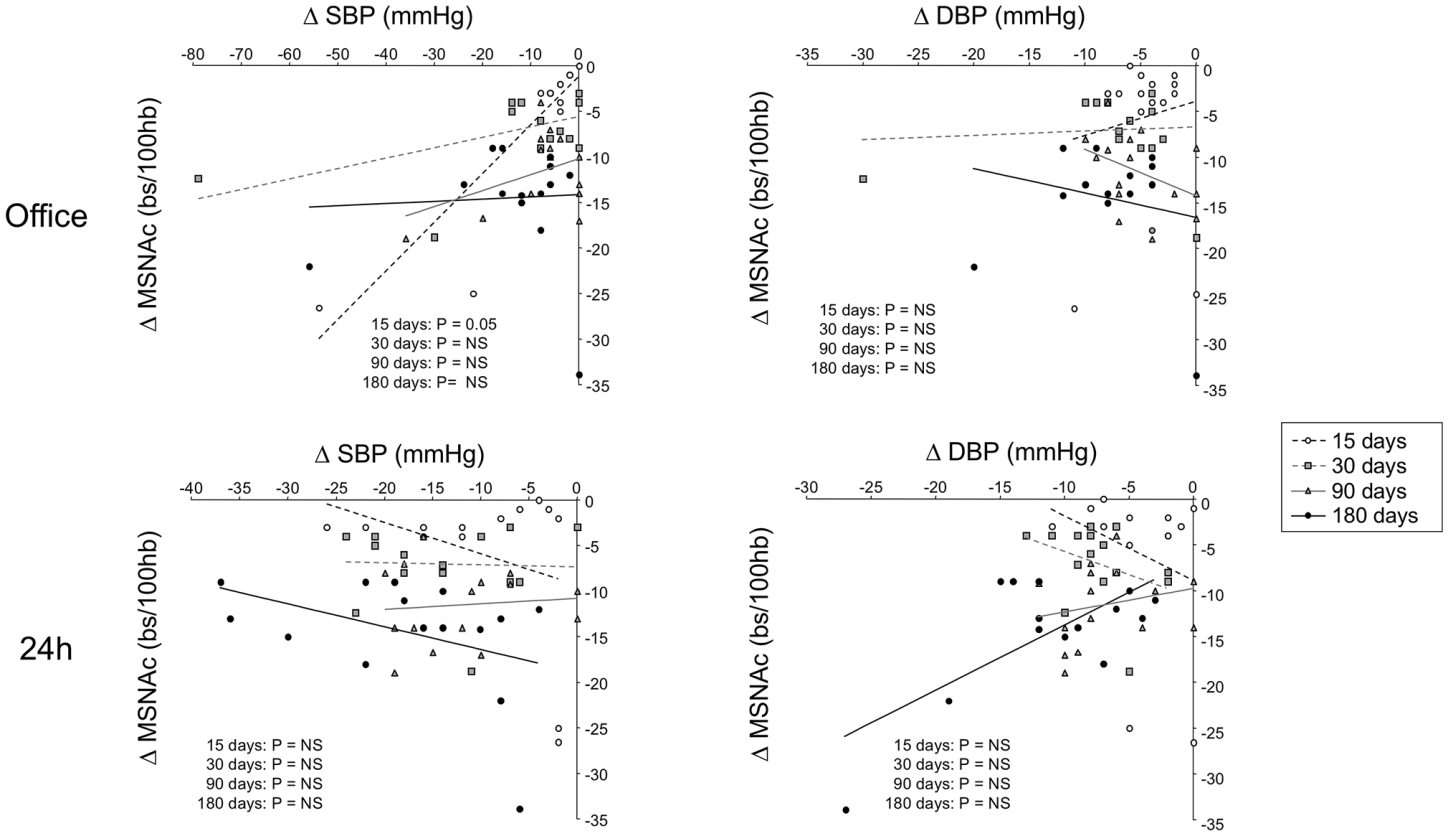
Lack of significant relationships between changes in office, ambulatory systolic (SBP) or diastolic (DBP) blood pressure values and the concomitant changes in muscle sympathetic nerve traffic [MSNA, expressed as burst incidence corrected for heart rate (bs/100 hb)] in 15 patients with drug-resistant hypertension 15, 30, 90 and 180 days after renal denervation. Note the lack of statistical significance, with only one casual exception (office SBP vs. MSNA changes 15 days after renal denervation). Individual changes in different time periods after renal denervation are shown. Figure adapted from data in Ref. [53]

To make the issue more complex, it should be emphasized that in microneurographic studies [51, 52], no significant relationship was detected in patients with drug-resistant hypertension between the reduction in clinic and 24-h ambulatory blood pressure values observed in different time periods following the procedure and the concomitant changes in muscle sympathetic nerve traffic (Fig. 3) [52].

Finally, no information is available on the interrelationships between the changes in sympathetic cardiovascular drive, blood pressure and renal function in chronic renal failure. Similarly, no data are available on the impact of bilateral renal nerve ablation on parasympathetic function in chronic kidney disease.

### Autonomic effects of baroreflex activation in chronic kidney disease

In both drug-resistant hypertension and congestive heart failure, evidence has shown that baroreflex activation via device-induced chronic electrical stimulation of the carotid baroreceptors triggers marked sympathoinhibitory effects, as assessed via the microneurographic recording of efferent postganglionic muscle sympathetic nerve traffic in the peroneal nerve [53, 54]. However, as compared to first-generation bilateral bipolar catheters, second-generation devices appear to exert less pronounced sympathomodulatory effects [55]. The sympathoinhibitory effects were associated with (and likely responsible for) the blood pressure reduction in drug-resistant hypertension [53]. In patients with congestive heart failure, it was also associated with an improvement in cardiac hemodynamic profile and New York Heart Association functional class [54]. No significant change in renal function was observed following device implantation in either drug-resistant hypertension or congestive heart failure cases [53, 54]

Only two studies so far have evaluated the effects of baroreflex activation therapy in patients with established chronic kidney disease [56, 57]. In the first study, in patients with drug-resistant hypertension and renal disease, the procedure prevented the deterioration of the renal function detected in the control-sham group during the 6-month follow-up [56]. In the second study, performed in patients with end-stage renal disease maintained on hemodialysis, the procedure enabled satisfactory blood pressure control to be achieved at 1-year follow-up [57]. Neither study provided any assessment of the sympathetic function following baroreflex activation therapy. However, they both reported some reduction in resting heart rate values after the procedure, a finding that may speak in favor of some decrease in the cardiac adrenergic drive (and concomitantly some increase in the parasympathetic function) in response to the intervention.

Altogether, the results of these studies call for future large-scale investigations aimed at testing the nephroprotective effects of baroreflex activation therapy in chronic renal failure. These studies should also allow us to clarify whether and to what extent the renal outcomes of the procedure are mediated not only by its blood pressure lowering effects but also by its sympathomodulatory effects.

## Perspectives and conclusions

In discussing the autonomic responses to the available therapeutic interventions for treating chronic kidney disease, several issues still remain unanswered. Three in particular deserve specific mention. First, the heart–kidney neurohumoral interactions may represent important targets in the near future for therapeutic interventions aimed at exerting neuromodulatory effects [58]. Second, future studies will allow us to clarify whether the pharmacologic, non-pharmacologic or device-based interventions described previously will be capable of restoring a normal autonomic function in patients with nephropathy. Although the available evidence is limited, an in-depth analysis of the results obtained with the different procedures mentioned above may suggest that multiple-drug combination treatment which includes compounds acting on the renin–angiotensin system and central sympatholytic agents may restore a “normal” sympathetic function in patients with chronic kidney disease, superposable to that described in healthy individuals [28]. Similar conclusions are achieved in analyzing the results of the 43-month follow-up study we performed in congestive heart failure patients after baroreflex activation therapy [59].

The final question has to do with the magnitude of the sympathoinhibitory effects we should obtain during treatment. Although we do not have any answer to this question at present, we should not forget that excessive sympathoinhibitory effects exerted by therapeutic interventions have been shown to produce deleterious effects on morbidity and mortality. This was demonstrated in the MOXonidine CONgestive Heart Failure (MOXCON) trial performed in advanced heart failure, employing too high a daily dose of moxonidine [60]. This was also shown in hypertensive patients recruited in the INternational VErapamil SR-Trandolapril STudy (INVEST), in which achievement of clinic heart rate values below 55 cardiac beats per minute during beta-blocking drug treatment favored a paradoxical increase in adverse events [61]. Future studies are thus needed in chronic renal failure patients to clarify the unsolved questions delineated above and to collect more detailed information on the autonomic effects of the therapeutic procedures currently employed in the treatment of chronic renal disease.

## References

[1] Esler M, Jennings G, Lambert G, Meredith I, Horne M, Eisenhofer G (1990). Overflow odf catecholamines neurotransmitters tothe circulation:source, fate and function. Physiol Rev.

[2] DiBona GF, Kopp UC (1997). Neural control of renal function. Physiol Rev.

[3] Schlaich MP, Socratous F, Hennebry S, Eikelis N, Lambert E, Straznicki N, Esler MD, Lambert GW (2009). Sympathetic activation in chronic renal failure. J Am Soc Nephrol.

[4] Grassi G, Biffi A, Seravalle G, Bertoli S, Airoldi F, Corrao G, Pisano A, Mallamaci F, Mancia G, Zoccali C (2021). Sympathetic nerve traffic overactivity in chronic kidney disease: a systematic review and meta-analysis. J Hypertens.

[5] Ewen S, Ukena C, Linz D, Schmieder RE, Bohm M, Mafhoud F (2013). The sympathetic nervous system in chronic kidney disease. Curr Hypertens Rep.

[6] Salman IM (2015). Cardiovascular autonomic dysfunction in chronic kidney disease: a comprehensive review. Curr Hypertens Rep.

[7] Grassi G, Bertoli S, Seravalle G (2012). Sympathetic nervous system: role in hypertension and in chronic kidney disease. Curr Opin Nephrol Hypertens.

[8] Durand AC, Jouve E, Delaroziere JC, Boucekine M, Izaaryene G, Crémades A, Mazouè F, Devictor B, Kahar A, Sambuc R, Brunet P, Gentile S (2018). End-stage renal disease treated in Provence-Aples Cote d’Azur: 12 years follow-up and forecast to the year 2030. BMC Nephrol.

[9] Thomson BJ, McAreavey D, Neilson JMM, Winney RJ, Ewing DJ (1991). Heart rate variability and cardiac arrhythmias in patients with chronic renal failure. Clin Autonom Res.

[10] Ranpuria R, Hall M, Chan CT, Unruh M (2008). Heart rate variability (HRV) in kidney failure: measurement and consequences of reduced HRV. Nephrol Dial Transplant.

[11] Penne EL, Neumann J, Klein IH, Oey PL, Bots ML, Blankestijn PJ (2009). Sympathetic hyperactivity and clinical outcome in chronic kidney disease patients during standard treatment. J Nephrol.

[12] Zoccali C, Mallamaci F, Parlongo S, Cutrupi S, Benedetto FA, Tripepi G, Bonanno G, Rapisarda F, Fatuzzo P, Seminara G, Cataliotti A, Tancanelli B, Malatino LS (2002). Plasma norepinephrine predicts survival and incident cardiovascular events in patients with end-stage renal disease. Circulation.

[13] Jassal S, Coulshed S, Douglas J, Stout R (1997). Autonomic neuropathy predisposing to arrhythmias in hemodialysis patients. Am J Kidney Dis.

[14] Hathaway DK, Cashion AK, Milstead EJ, Winsett P, Cowan PA, Wicks MN, Gaber AO (1998). Autonomic dysfunction in patients awaiting renal transplantation. Am J Kidney Dis.

[15] Williams B, Mancia G, Spiering W, Agabiti Rosei E, Azizi M, Burnier M, Clement DL, Coca A, de Simone G, Dominiczak A, Kahan T, Mahfoud F, Redon J, Ruilope L, Zanchetti A, Kerins M, Kjeldsen SE, Kreutz R, Laurent S, Lip GYH, McManus R, Narkiewicz K, Ruschitzka F, Schmieder RE, Shlyakhto E, Tsioufis C, Aboyans V, Desomais I (2018). 2018 ESC/ESH Guidelines for the management of arterial hypertension: the task force for the management of arterial hypertension of the European Society of Cardiology and the European Society of Hypertension. Eur Heart J.

[16] Tory K, Horvath E, Suveges Z, Fekete A, Sallay P, Berta K, Szabó T, Szabó AJ, Tulassay T, Reusz GS (2004). Effect of propranolol on heart rate variability in patients with endstage renal disease: a double-blind, placebo-controlled, randomized crossover pilot trial. Clin Nephrol.

[17] Sato R, Mizuno M, Miura T, Kato Y, Watanabe S, Fuwa D, Ogiyama Y, Tomonari T, Ichikawa T, Shirasawa Y, Ito A, Ota K, Yoshida A, Fikuda M, Kimura G (2013). Angiotensin receptor blockers regulate the synchronization of circadian rhythms in heart rate and blood pressure. J Hypertens.

[18] Ondocin PT, Narsipur SS (2006). Influence of angiotensin converting enzyme inhibitor treatment on cardiac autonomic modulation in patients receiving haemodialysis. Nephrology.

[19] Narsipur SS, Srinivasan B, Singh B (2011). Effect of simvastatin use on autonomic function in patients with end-stage renal disease. Cardiovasc Hematol Disord Drug Targets.

[20] Ligtenberg G, Blankestijn PJ, Oey PL, Klein IH, Dijkhorst-Oei LT, Boomsma F, Wieneke GH, Van Huffelen AC, Koomans HA (1999). Reduction of sympathetic hyperactivity by enalapril in patients with chronic renal failure. N Engl J Med.

[21] Neumann J, Ligtenberg G, Klein IH, Boer P, Oey PL, Koomans HA, Blankestijn PJ (2007). Sympathetic hyperactivity in hypertensive chronic kidney disease patients is reduced during standard treatment. Hypertension.

[22] Grassi G, Seravalle G, Esler MD (2021). Sympathomodulation in heart failure: from drugs to devices. Int J Cardiol.

[23] Siddiqi L, Oey PL, Blankestijn PJ (2011). Aliskiren reduces sympathetic nerve activity and blood pressure in chronic kidney disease patients. Nephrol Dial Transplant.

[24] Siddiqi L, Joles JA, Oey PL, Blankestijn PJ (2011). Atorvastatin reduces sympathetic activity in patients with chronic kidney disease. J Hypertens.

[25] Zoccali C, Seravalle G, Grassi G (2011). Sympathoinhibitory effects of statins in chronic kidney disease: are they clinically relevant?. J Hypertens.

[26] Levitan D, Massry SG, Romoff M, Campese VM (1984). Plasma catecholamines and autonomic nervous system function in patients with early renal insufficiency and hypertension: effect of clonidine. Nephron.

[27] Vonend O, Marsalek P, Russ H, Wulkow R, Oberhauser V, Rump LC (2003). Moxonidine treatment of hypertensive patients with advanced renal failure. J Hypertens.

[28] Neumann J, Ligtenberg G, Oey L, Koomans HA, Blankestijn PJ (2004). Moxonidine normalizes sympathetic hyperactivity in patients with eprosartan-treated chronic renal failure. J Am Soc Nephrol.

[29] Ewing DJ, Wunney R (1975). Autonomic function in patients with chronic renal failure on intermittent hemodialysis. Nephron.

[30] Converse RL, Jacobsen TN, Toto RD, Jost CM, Cosentino F, Fouad-Tarazi F, Victor RC (1992). Sympathetic overactivity in patients with chronic renal failure. N Engl J Med.

[31] Rosansky SJ, Rhinehart R, Whittman D, Menachery SJ (1995). Comparison of autonomic function using Valsalva ratio and 30:15 postural ratio prior to and after haemodialysis treatment. Clin Autonom Research.

[32] Zucchelli P, Santoro A, Sturani A, Degli Esposti E, Chiarini C, Zuccalà A (1984). Effects of haemodialysis and hemofiltration on autonomic control of circulation. Trans Am Soc Art Intern Organs.

[33] Agarwal A, Anand S, Sakhuja V, Chugh KS (1991). Effect of dialysis and renal transplantation on autonomic dysfunction in chronic renal failure. Kidney Int.

[34] Grassi G, Parati G, Pomidossi G, Giannattasio C, Casadei R, Bolla G, Saino A, Casati S, Graziani G, Mancia G (1987). Effects of haemodialysis and kidney transplantation on carotid and cardiopulmonary baroreflexes in uraemic patients. J Hypertens.

[35] Notarius CF, Morris BL, Floras JS (2009). Dissociation between reflex sympathetic and forearm vascular responses to lower body negative pressure in heart failure patients with coronary artery disease. Am J Physiol.

[36] Wiese F, London G, Panier BM, Guerin AP, Elghozi JL (1995). Effects of hemodialysis on cardiovascular rhytms in end-stage renal disease. Kidney Int.

[37] Chan CT, Jain V, Picton P, Pierratos A, Floras JS (2005). Nocturnal hemodialysis increases baroreflex sensitivity and compliance and normalizes blood pressure of hypertensive patients with end-stage renal disease. Kidney Int.

[38] Chan CT, Harvey PJ, Picton P, Pierratos A, Miller JA, Floras JS (2003). Short-term blood pressure, noradrenergic and vascular effects of nocturnal home hemodialysis. Hypertension.

[39] Zilch O, Vos PF, Oey L, Cramer MJM, Ligtenberg G, Kroomans HA, Blankesijn PJ (2007). Sympathetic hyperactivity in haemodialysis patients is reduced by short daily haemodialysis. J Hypertens.

[40] Rubinger D, Sapoznikov D, Pollak A, Popovtzer MM, Luria MH (1999). Heart rate variability during chronic hemodialysis and after renal transplantation: studies in patients without and with systemic amyloidosis. J Am Soc Nephrol.

[41] Kurata C, Uehara A, Ishikawa A (2004). Improvement of cardiac sympathetic innervation by renal transplantation. J Nucl Med.

[42] Hausberg M, Kosch M, Harmelink P, Barenbrock M, Hohage H, Kisters K, Dietl KH, Rahn K (2002). Sympathetic nerve activity in end-stage renal disease. Circulation.

[43] Scherrer U, Vissing SF, Morgan BJ, Rollins JA, Tindall RS, Ring S, Hanson P, Mohanty PK, Victor R (1990). Cyclosporine-induced sympathetic activation and hypertension after heart transplantation. N Engl J Med.

[44] Siddiqi L, Joles JA, Grassi G, Blankestijn PJ (2009). Is kidney ischemia the central mechanism in parallel activation of the renin and the sympathetic system?. J Hypertens.

[45] Mahfoud F, Bohm M, Schmieder R, Narkiewicz K, Ewen S, Ruilope L, Schlaich M, Williams B, Fahy M, Mancia G (2019). Effects of renal denervation on kidney function and long-term outcomes: 3-year follow-up from the Global SYMPLICITY Registry. Eur Heart J.

[46] Ott C, Mahfoud F, Schmid A, Toennes SW, Ewen S, Ditting T, Veelke R, Ukena C, Uder M, Bohm M, Schmieder R (2015). Renal denervation preserves renal function in patients with chronic kidney disease and resistant hypertension. J Hypertens.

[47] Hering D, Marusic P, Duvak J, Sata Y, Head GA, Burrows S, Walton AS, Esler MD, Schlaich MP (2017). Effect of renal denervation on kidney function in patients with chronic kidney disease. Int J Cardiol.

[48] Scalise F, Sole A, Singh G, Sorropago A, Sorropago G, Ballabeni C, Maccario M, Vettoretti S, Grassi G, Mancia G (2020). Renal denervation in patients with end-stage renal disease and resistant hypertension on long-term hemodialysis. J Hypertens.

[49] De Beus E, de Jager R, Joles JA, Grassi G, Blankestijn PJ (2014). Sympathetic activation secondary to chronic kidney disease: therapeutic target for renal denervation?. J Hypertens.

[50] Hoye NA, Wilson LC, Wilkins GT, Jardine DL, Putt TL, Samaranayaka A, Schollum JBW, Walker RJ (2017). Endovascualr renal denervation in end-stage kidney disease patients: cardiovascular protection—a proof-of-concept study. Kidney Int Rep.

[51] Brinkmann J, Heusser K, Schmidt B, Menne J, Klein G, Bauersachs J, Haller H, Sweep FC, Diedrich A, Jordan J, Tank J (2012). Catheter-based renal nerve ablation and centrally-generated sympathetic activity in difficult-to-control hypertensive patients: prospective case series. Hypertension.

[52] Grassi G, Seravalle G, Brambilla G, Trabattoni D, Cuspidi C, Corso R, Pieruzzi F, Genovesi S, Stella A, Facchetti R, Spaziani D, Bartorelli A, Mancia G (2015). Blood pressure responses to renal denervation precede and are independent of the sympathetic and baroreflex effects. Hypertension.

[53] Jordan J (2017). Device-based approaches for the treatment of arterial hypertension. Curr Hypertens Rep.

[54] Gronda E, Seravalle G, Brambilla G, Costantino G, Casini A, Alsheraei A, Lovett EG, Mancia G, Grassi G (2014). Chronic baroreflex activation effects on sympathetic nerve traffic, baroreflex function and cardiac haemodynamics in heart failure. A proof-of-concept study. Eur J Heart Fail.

[55] Heusser K, Tank J, Brinkmann J, Menne J, Kaufeld J, Linnenweber-Held S, Beige J, Wilhelmi M, Diedrich A, Haller H, Jordan J (2016). Acute response to unilateral unipolar electrical carotid sinus stimulation in patients with resistant arterial hypertension. Hypertension.

[56] Alnima T, de Leeuw PW, Tan FES, Kroon AA, for the Rheos Pivotal trial investigators (2013). Renal responses to long-term carotid baroreflex activation therapy in patients with drug-resistant hypertension. Hypertension.

[57] Wallbach M, Lehnig LY, Schroer C, Hasenfuss G, Muller GA, Wacter R, Kozioleck MJ (2014). Impact of baroreflex activation therapy on renal function-a pilot study. Am J Nephrol.

[58] Zoccali C, Tripepi G, Dounousi E, Mallamaci F (2014). Chronic kidney disease /CKD) as a systemic disease:whole body autoregulation and inter-organ crosstalk. Kidney Blood Press Res.

[59] Dell'oro R, Gronda E, Seravalle G, Costantino G, Alberti L, Baronio B, Staine T, Vanoli E, Mancia G, Grassi G (2017). Restoration of normal sympathetic neural function in heart failure following baroreflex activation therapy. Final 43-month study report. J Hypertens.

[60] Cohn JN, Pfeffer MA, Rouleau J, Sharpe N, Swedberg K, Straub M, Wiltse C, Wright TJ, MOXCON Investigators (2013). Adverse mortality effect of central sympathetic inhibition with sustained-release moxonidine in patients with heart failure (MOXCON). Eur J Heart Fail.

[61] Kolloch R, Legler UF, Champion A, Cooper-Dehoff RM, Handberg E, Zhou Q, Pepine CJ (2008). Impact of resting heart rate on outcomes in hypertensive patients with coronary artery disease: findings from the INternational VErapamil-SR/trandolapril STudy (INVEST). Eur Heart J.

